# Long non-coding RNA H19, a novel therapeutic target for pancreatic cancer

**DOI:** 10.1186/s10020-020-00156-4

**Published:** 2020-04-09

**Authors:** Jing Wang, Lei Zhao, Kun Shang, Fang Liu, Juanjuan Che, Huihui Li, Bangwei Cao

**Affiliations:** 1 Department of Oncology, Beijing Friendship Hospital, Capital Medical University, #95 Yong An Road, Beijing, Xicheng District China; 2 Yale School of Medicine, New Haven, CT USA; 3 Department of Cardiology, Chaoyang Hospital, Capital Medical University, Beijing, Chaoyang District China

**Keywords:** Long non-coding RNA, H19, pancreatic ductal adenocarcinoma, Proliferation, Therapeutic target

## Abstract

Pancreatic ductal adenocarcinoma (PDAC) is an aggressive malignancy with high mortality, which threats peoples’ health. Unfortunately, the pathogenesis of PDAC remains unclear. Recent studies have indicated that long non-coding RNAs (lncRNAs) can regulate the development and progression of malignant tumors through varying mechanisms. LncRNA H19 has a unique expression profile and can act as a sponger of specific miRNAs to regulate the pathogenic process of many diseases, including PDAC and several other types of cancers. Here, we review the research approaches to understanding the regulatory role of H19 and potential mechanisms in the progression of PDAC and other types of cancers and diseases. These studies suggest that H19 may be a novel therapeutic target for PDAC and our findings may open new revenues for scientific researches and development of valuable therapies for these diseases in the future.

## Background

PDAC is one of the leading causes for human cancer-related death worldwide. Its incidence is increasing and there are about more than 450,000 new cases that cause 430,000 deaths yearly in the world (Rawla et al. 2019). Currently, patients with PDAC are usually treated with surgical resection, chemotherapy, and radiotherapy and they may need one or combined therapeutic strategies, dependent on the location and stages of PDAC. However, there is no new effective therapy available for treatment of PDAC patients.

Previous studies have shown that bacteria, genetic and environmental factors, including lncRNAs can regulate the development and progression of PDAC. Therefore, further understanding of how these factors regulate the development and progression of PDAC may uncover new therapeutic targets for intervention of PDAC.

## Introduction

The pancreas is an important endocrine and exocrine organ for digestion of foods and its islets are composed of endocrinal cells that secrete insulin, glucagon, somatostatin and pancreatic polypeptide to regulate glucose tolerance and other functions. Pancreatic cancers, particularly pancreatic ductal adenocarcinoma (PDAC), are very aggressive and have a high mortality rate due to the lack of specific therapies and understanding their pathogenesis. It is notable that long non-coding RNAs (lncRNAs) can regulate many processes, including the development and progression of PDAC, and recent studies have shown that lncRNA H19 is a critical regulator of PDAC (Yoshimura et al. 2018; Sasaki et al. 2018; Ma et al. 2016; Ma et al. 2014). Here, we review the updated information on the function and potential mechanisms of lncRNA H19 in regulating the development and progression of PDAC as well as other diseases.

### Pancreatic cancers

Pancreatic cancers are heterogeneous and can be stratified into pancreatic exocrine and neuroendocrine tumors, according to their originating cells. Pancreatic exocrine cancers are the majority of pancreatic cancers and can be classified into PDAC, acinar cell carcinoma, intra-ductal papillary-mucinous neoplasm (IPMN) and mucinous cystic neoplasm with an invasive adenocarcinoma as well as rare colloid cancer and solid pseudopapillary tumor. The PDAC accounts for about 85% of pancreatic cancers, and is one of the leading causes for human cancer-related death worldwide (Rawla et al. 2019; Carioli et al. 2019). Currently, there is no new effective therapy for the treatment of PDAC patients besides traditional surgical resection, chemotherapy and radiotherapy. Erlotinib targeted therapy has been used for the treatment of PDAC, but its long-term benefit is limited (Li et al. 2014). These, together with vague unexplained symptoms, difficult for early diagnosis, a low surgical rate for resection of the tumors and high recurrence and metastatic rates, contribute to low 5-year survival and high mortality of PDAC patients. More importantly, although many risk factors, such as smoking, alcohol abuse, obesity, dietary factors and exposure to toxic substances, are associated with the development and progression of pancreatic cancer, the pathogenesis of PDAC remains unclear. Previous studies have shown that bacteria, genetic and environmental factors, including lncRNAs, can regulate the development and progression of PDAC (Gentiluomo et al. 2019; Campa et al. 2019; Karpinski 2019). Therefore, further understanding of how these factors regulate the development and progression of PDAC may uncover new therapeutic targets for intervention of PDAC.

### LncRNAs, H19 and their basic functions

**Fig. 1:**
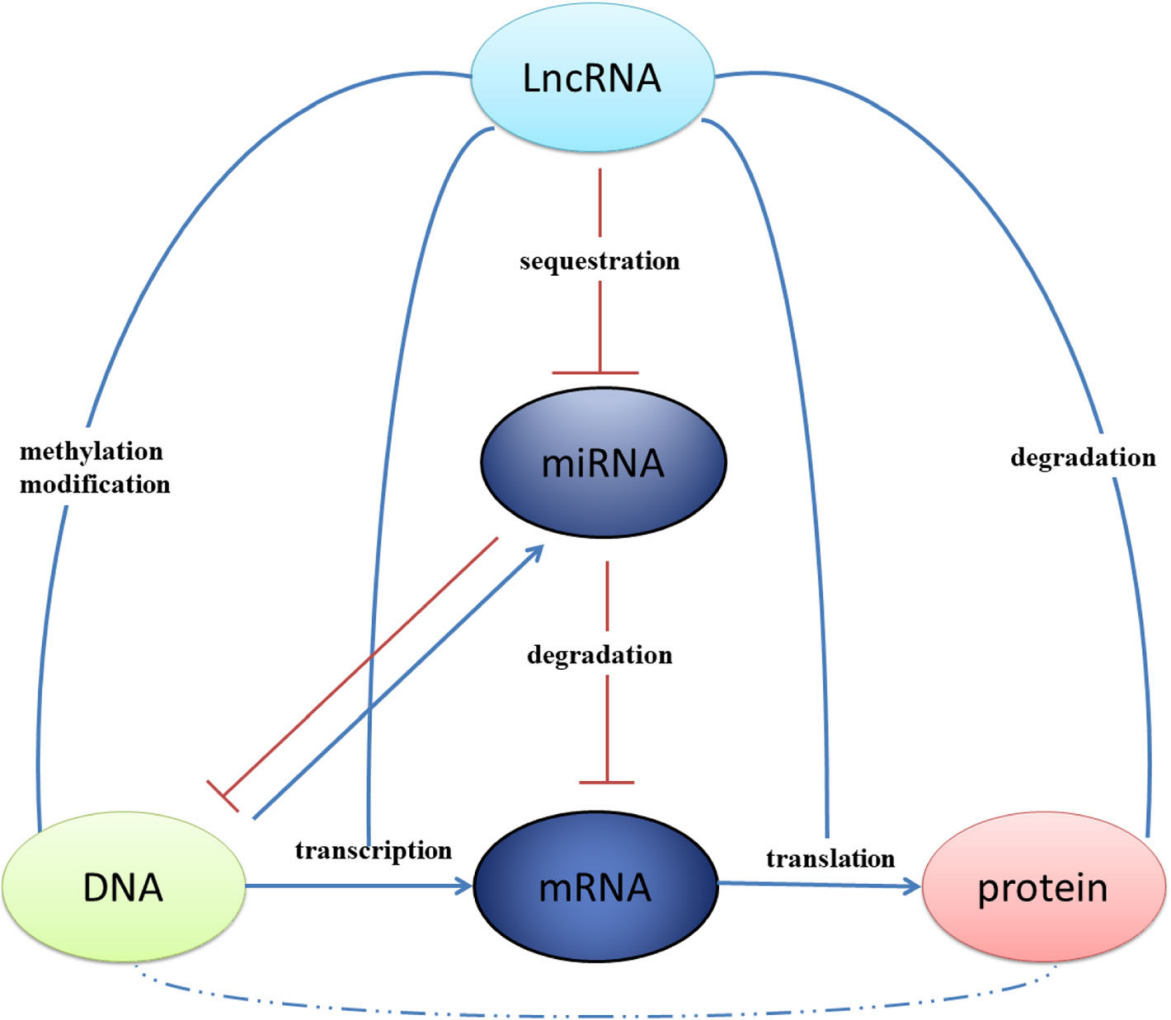
The lncRNA-miRNA-mRNA regulation network

LncRNAs are abundant RNA transcripts of > 200 nt, transcribed by RNA polymerase II from different regions of a genome (Jarroux et al. 2017). However, only in less than 200 lncRNAs, their basic functions were identified (Unfried et al. 2019). LncRNAs can be present in the cytoplasm and/or nucleus of cells and function to act as an epigenetic factor or sponger to regulate gene expression by binding to miRNA, mRNA, and DNA. Furthermore, lncRNAs can act as a signaling, decoy, guiding or scaffold molecule by binding to transcription factors and co-activators to regulate protein activity and signaling. Some lncRNAs may encode proteins. Their basic functions and functional mechanisms are summarized in Fig. 1.

**Fig. 2:**
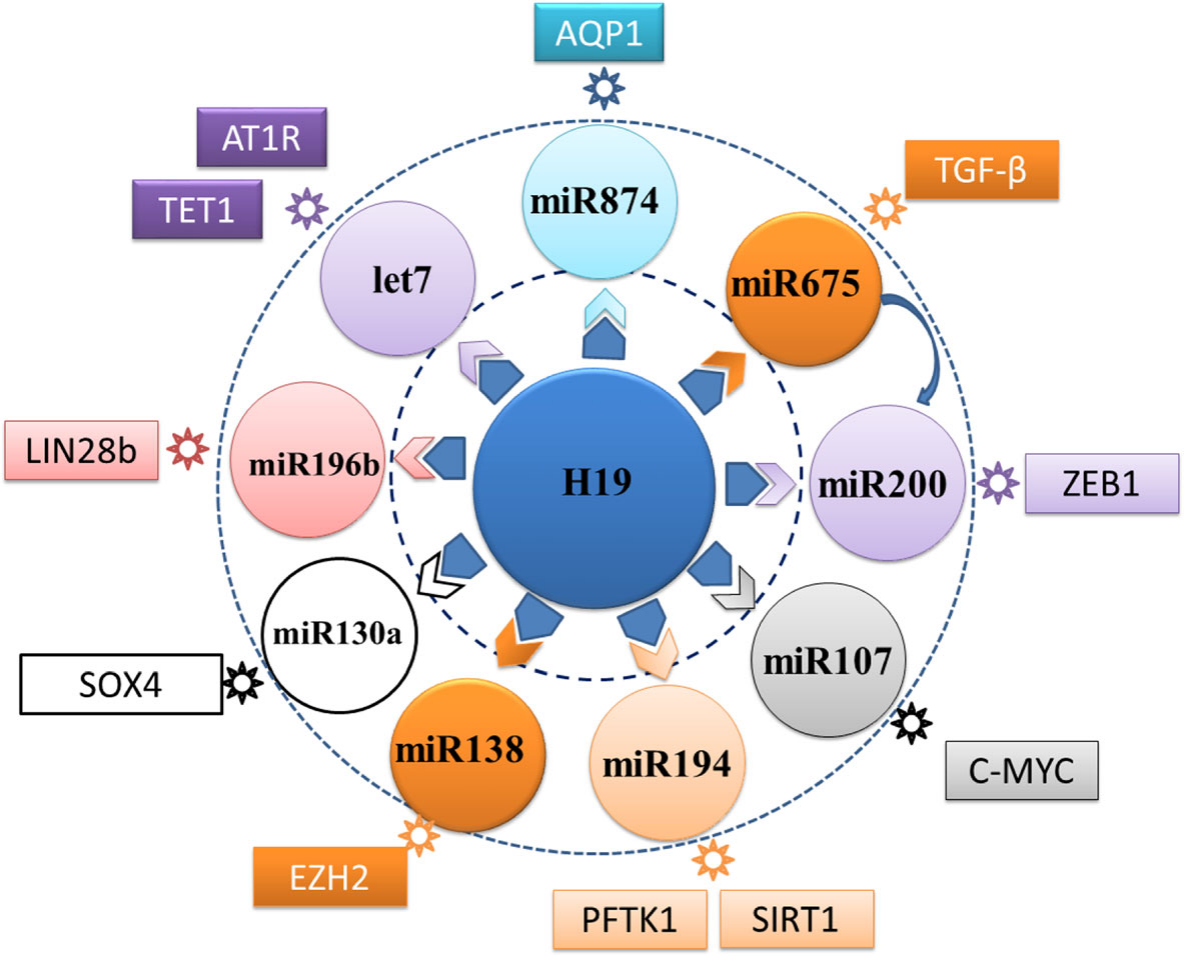
The major functions of H19

The H19 is an imprinted gene in the 11p15.5 region and is expressed exclusively from the maternal allele usually in the early stages of embryogenesis, but declined postnatally. The H19 encodes for 2.3 kb RNA and is transcribed by RNA polymerase II, spliced and polyadenylated. LncRNA H19 is exported to the cytoplasm from the nucleus. H19 expression is regulated by neighboring *Igf2* gene and transcription factors in the C/EBP family. Furthermore, H19 expression is also regulated by the imprinted control region, CTCF insulation, different enhancers, and matrix attachment regions (Gabory et al. 2006). Current studies have shown that H19 predominantly acts as a sponger of miRNAs, including miRNA138 (Hong et al. 2018), miRNA200 (Liu et al. 2015) and others to enhance the expression of their targeted genes in a context and cell-specific manner, dependent on the cell type and status in different types of cancers. Actually, H19 silencing increased miR-138 expression and inhibited the proliferation, and invasion of oral squamous cell carcinoma cells in vitro and in vivo by reducing enhancer of zeste homolog 2 (EZH2) expression, which were attenuated by miR-138 silencing (Hong et al. 2018). Zhang L et al. (Zhang et al. 2013) found that H19 was associated with the protein complex hnRNP U/PCAF/RNAPol II to increase miR-200 expression by enhancing histone acetylation. Such data indicate that H19 can alter the miR-200 pathway, contributing to the mesenchymal epithelial transition (MET) process and to the suppression of tumor metastasis. The major functions of H19 are summarized in Fig. 2.

H19 has diverse functions in regulating different processes in varying types of cells. Functionally, H19 mainly acts as a sponger or ceRNA of its targeted miRNAs, such as miR-874, miR-675, miR-200, miR-107, miR194, miR-130a, miR196b, let-7b and others to modulate their targeted gene expression, including AQP1, PPRα, ZEB1, cMyc, PFTK1/SIRT1, SOX4, LIN28b, TET/AT1R and others in varying types of cells (Zhang et al. 2013; Fang et al. 2018; Luo et al. 2019; Gregory et al. 2008; Song et al. 2017; Cui et al. 2015; Sun et al. 2019; Han et al. 2018; Hu et al. 2018; Ren et al. 2018; Su et al. 2018; Chen et al. 2019; Kallen et al. 2013). In addition, H19 can also bind to ZEB1 to enhance EpCAM expression, contributing to the pathogenesis of cholestatic liver fibrosis (Song et al. 2017). Apparently, H19 can target many miRNAs to regulate the broad biological processes (see detailed discussion below). However, there is no information on whether H19 can participate in epigenetic regulation, directly target coactivators to regulate their function or encode a protein.

### H19 in PDAC

Many studies have confirmed that H19 is closely associated with the development and progression of PDAC (Yoshimura et al. 2018; Sasaki et al. 2018; Sun et al. 2019). First, salivary lncRNA H19 levels are similar in both PDAC and non-tumor patients although the levels of several other lncRNAs are significantly different between them (Xie et al. 2016). H19 and *E2F-1* expression are up-regulated in human PDAC tissues (Ma et al. 2016). Mouse models of xenograft PDAC revealed that H19 silencing significantly inhibited the growth of implanted PDCA and reduced their volumes and weights (Yoshimura et al. 2018), In contrast, H19 over-expression accelerated the growth of implanted PDAC in vivo (Yoshimura et al. 2018). Interestingly, H19 silencing down-regulated E2F-1 expression in PANC-1 and T3M4 cells while H19 over-expression up-regulated E2F-1 expression in COLO357 and CAPAN-1 cells (Ma et al. 2016). Furthermore, H19 may act as a sponger of miR-675 to promote PDAC cell proliferation by enhancing E2F-1 expression (Ma et al. 2018). Ma et al. (Ma et al. 2018) found that the levels of H19 expression were inversely correlated significantly with miR-675 in PDAC tissues. The levels of serum miR-675 decreased before surgical resection, but were restored in PDAC patients after surgery. While miR-675 over-expression decreased cell viability and clonogenicity by inducing cell cycle arrest in S phase in ASPC-1 and PANC-1 cells miR-675 silencing restored the proliferation of PDAC cells, which had been inhibited by H19 silencing. Bioinformatics and luciferase activity assay indicated that miR-675 targeted E2F-1 while miR-675 silencing restored the E2F-1 protein expression in the H19-silenced PDAC cells. Therefore, H19 acts as an oncogenic factor by targeting miR-675 in PDAC and the H19/miR-675/E2F-1 regulatory loop is crucial for regulating the growth and progression of PDAC.

H19 can promote the metastasis of PDAC. First, H19 and *PFTK1* expression were co-up-regulated in PDAC of the TCGA database (Sun et al. 2019). While H19 silencing inhibited the proliferation and migration of PDAC cells, PFTK1 over-expression restored the H19 silencing-inhibited proliferation and migration of PDAC cells. Furthermore, PFTK1 expression was negatively correlated with miR-194 expression in PDAC from the TCGA and other data resources. Further luciferase reporter and RNA precipitation assays demonstrated that H19 acted as a sponger of miR-194 that targeted the *PFTK1* mRNA in PDAC cells. It is well known that PFTK1 is crucial for the Wnt/β-catenin signaling, which can regulate the growth and metastasis of PDAC. Actually, miR-194 over-expression decreased PFTK1 protein expression and attenuated the Wnt/β-catenin signaling, PDAC cell proliferation and invasion. Similarly, H19 expression was significantly up-regulated in the metastatic PDAC, related to non-metastatic PDAC, and H19 silencing inhibited the PDAC cell invasion in vitro and PDAC metastasis in vivo (Sasaki et al. 2018; Ma et al. 2014). While *H19* over-expression significantly enhanced PDAC cell adhesion *H19 silencing reduced their adhesion*. Moreover, H19 silencing inhibited the hepatic metastasis of PDAC by significantly reducing the sizes and numbers of metastatic PDAC in the liver of NOD/scid mice following observation for 11 weeks (Yoshimura et al. 2018). In addition, H19 promoted PDAC stem-like cell adhesion by up-regulating the expression of integrin and CD24, which was abrogated by anti-integrin β1. Moreover, H19 enhanced the high mobility group AT-hook 2 (HMGA2)-induced EMT process of PDAC cells by antagonizing let-7 to enhance the invasion and migration of PDAC cells. Such findings indicate that H19 positively regulates the invasion of PDAC stem-like cells, contributing to the metastasis of PDAC. Together, H19 sponges different miRNAs to promote the growth and metastasis of PDAC although the precise mechanisms underlying the action of H19 remain to be further explored.

H19 may be a new therapeutic target for treatment of PDAC. Given that H19 expression is up-regulated in malignant cancer, scientists in the Hebrew University of Jerusalem developed a plasmid (BC-19, DTA-H19) for the H19 promoter-controlled diphtheria toxin A (DTA) expression. Scaiewicz et al. (Scaiewicz et al. 2010) found that treatment with DTA-H19 inhibited PDAC growth in different animal models by reducing tumor volumes of 25–50% and prevented PDAC metastasis. Furthermore, sequential treatment with BC-H19 and gemcitabine achieved better anti-tumor efficacy than gemcitabine alone in an animal model of PDAC (Scaiewicz et al. 2010). Following implantation with PDAC cells into NOD/scid mice, treatment with 50 μg DTA-H19 at 7 and 9 days post inoculation significantly reduced the size of PDAC tumors by near 50% compared with the control mice receiving the same dose of Luc-H19 (Scaiewicz et al. 2010). The results from an early clinical trial revealed that intratumoral administration of BC-H19 stabilized the progression of unresectable PDAC and combination with subsequent chemotherapies achieved the resection of PDAC and partial responses in 5 out of 9 patients (Hanna et al. 2012). This trial also demonstrated that endoscopic ultrasound (EUS) or CT-guided intratumoral administration of 8 mg BC-H19 twice per week was relatively safe in those patients. Currently, scientists have developed a BC-H19/polyethylenimine complex to seek stable H19-drived DTA expression, which is being tested in other types of malignancies. Although the early studies brought promising the success of H19-based therapies for PDAC may be far away from using in clinical practice.

### H19 in malignant tumors of other organs

H19 expression is up-regulated in several types of malignant tumors in humans and crucial for the pathogenesis and prognosis of several cancers. First, the A/A homozygous genotype of rs217727 SNP in the H19 was significantly associated with an increased lung cancer risk (odds ratio (OR) = 1.661, 95% confidence interval (CI) = 1.155 to 2.388, *P* = 0.006). Furthermore, the A/A genotype had a higher risk of lung cancer than those of G/G in the squamous cell carcinoma (OR = 2.022, *P* = 0.004) and adenocarcinoma (OR = 1.606, *P* = 0.045) subgroups (Li et al. 2018). Similarly, the A allele of rs2839698 SNP in the H19 is significantly associated with an increased risk for colorectal cancer (CRC) with an OR of 1.20 (Li et al. 2016a). Second, H19 expression is up-regulated in human lung cancers, gastric cancer, CRC, breast cancer, ovary cancer and glioma (Hu et al. 2018; Li et al. 2018; Yoruker et al. 2018; Dai et al. 2019; Si et al. 2019; Amit and Hochberg 2012) and associated with poor prognosis in these types of cancers. These indicate that H19 acts as an oncogenic factor to promote the progression of these cancers in humans. Actually, H19 over-expression promoted the malignant behaviors, including aggressive proliferation, clonogenicity, EMT process, migration, invasion in vitro and rapid growth and metastasis in vivo, of different types of cancer cells while H19 silencing mitigates their malignant behaviors (Sun et al. 2019; Ma et al. 2018; Si et al. 2019; Peng et al. 2017; Zhang et al. 2018; Jiang et al. 2016). Mechanistically, H19 acts as a sponger of miR-19 to enhance STAT3 expression in lung cancer and it may also target miR-107, miR-21, miR-196b and miR-484 in different types of lung cancers. Furthermore, H19 can also participate in exosome formation to spread resistance to gefitinib and other tyrosine kinase inhibitors (TKIs) (Sun et al. 2018). H19 sponges miR-223p to enhance Snail expression and EMT process in gastric cancer (Gan et al. 2019) while combined detections of H19, MEG3 and miR-675-5p have been demonstrated to effectively distinguish gastric cancer from non-tumor samples with a specificity or sensitivity of > 85% in the clinic (Ghaedi et al. 2019). H19 targets miR-138 to enhance SOX4 expression, leading to enhanced EMT and metastasis in breast cancers and glioma (Si et al. 2019) and sponges both miR-138 and miR-194-5-p to promote high mobility group A (HMGA1) expression, EMT and metastasis in CRC (Yang et al. 2017). Interestingly, H19 can target miR-324-5p to increase pyruvate kinase M2 (PKM2) expression and glucose metabolism, and support the growth of ovary cancer (Zheng et al. 2018). Moreover, H19 is a biomarker for diagnosis of cholangiocarcinoma with high sensitivity and specificity (Han et al. 2018) and H19 can sponge Let-a/b, miR-372 and miR-373 to enhance IL-6 production, CXCR expression and tumor-related inflammation, and malignant behaviors of cholangiocarcinoma (Wang et al. 2016). Collectively, H19 acts as an oncogenic sponger to target specific miRNAs and enhance their targeted protein expression, promoting the progression of different types of cancers. The targeted miRNAs by H19 depend on the origin of the malignant tumors. Currently, it is unclear why the same H19 targets different miRNAs in different types of cancers.

Cancer stem cells (CSCs) can self-renew and differentiate into different types of the tumor cells and be associated with drug resistance, recurrence and metastasis of cancers (Wang et al. 2019). H19 is one of the lncRNA regulators for CSCs and its expression is up-regulated in CSC-like cells from several types of cancers (Jiang et al. 2016). H19 can sponge miR-200a to promote the stemness of CSCs (Liu et al. 2015). Our previous study and those of others have shown that H19 positively regulates the stemness and self-renewal of breast CSCs by anchoring let-7 to enhance LIN28 expression and the Wnt/β-catenin signaling while the function of H19 can be suppressed by let-7 to form a negative feedback loop (Peng et al. 2017; Wang et al. 2019). In addition, H19 also regulates drug resistance of several types of malignant tumors. H19 over-expression increases resistance to bortezomib by targeting miR-29b-39 to increase myeloid cell leukemia **1 (**MCL-1) expression in multiple myeloma cells (Pan et al. 2019) and to cisplatin by targeting miR-106b-5p to enhance testis development-related 1 (TDRG1) expression in seminoma cells (Wei et al. 2018). However, it is unclear whether H19 can regulate the differentiation, drug resistance and metastasis of CSCs through other miRNAs and/or other mechanisms.

### H19 in other diseases

H19 is an important regulator of the pathogenic process of many diseases and its regulatory functions have been extensively discussed (Liu et al. 2019; Aalijahan and Ghorbian 2019). Briefly, H19 can target miR-874 to enhance Aquaporin 1 expression to inhibit the lipopolysaccharide (LPS)-induced sepsis and myocardial dysfunction in animals (Fang et al. 2018). H19 also regulates the pathogenesis of osteoarthritis, myositis, and atherosclerosis (Steck et al. 2012; Hamann et al. 2017; Kumar et al. 2019). The precise role of H19 during the inflammatory process may depend on a disease model. For example, H19 appears to enhance inflammation by targeting miR-130b during the process of atherosclerosis because H19 silencing significantly mitigates TNF-α and IL-1β production in rodents. Similarly, H19 may promote the pathogenesis of pulmonary arterial hypertension (PAH) by targeting let-7b to enhance angiotensin II receptor type 1 (AT1R) expression and smooth muscular cell proliferation (Su et al. 2018). H19 can modulate the pathogenesis of ischemic stroke by enhancing the NF-kB signaling and pro-inflammatory cytokine production probably by regulating the miR-675/peroxisome proliferator-activated receptor (PPAR) α axis or microglial functions in rodents (Luo et al. 2019; Bao et al. 2018; Wang et al. 2017). Similarly, H19 participates in the pathogenic process of intestinal inflammation and it also promotes the regeneration of intestinal mucosal cells by inhibiting P53, miR-34a and let7 expression (Geng et al. 2018). In contrast, H19 can also reduce oxidative stress, inflammation and apoptosis of cardiomyocytes by maintaining miR-675 expression to down-regulate voltage-dependent anion-selective channel 1 (VDAC1) expression (Li et al. 2016b). In addition, H19 may negatively regulate the proliferation of corneal epithelial cells and alternation of its expression is associated with Russell-Silver and Beckwith-Wiedemann syndromes (Klein et al. 2016). Thus, H19 regulates inflammation, dependent on the environment.

Inflammation is associated with tissue fibrosis. H19 expression is up-regulated in the TGF-β1 or bleomycin-induced pulmonary fibrotic tissues and H19 targets miR-196a to enhance type I collagen alpha 1 (COL1A1) expression in the fibrotic lung tissues (Lu et al. 2018). Similarly, H19 expression is also up-regulated and enhances cholestatic liver fibrosis by promoting bile duct epithelial cell proliferation and enhancing epithelial cell adhesion molecule (EpCAM) expression to recruit pathogenic T cells (Song et al. 2017). This effect may be attributed to the direct interaction of H19 with e-box binding homologous box 1 (ZEB1) to reduce its inhibition on EpCAM expression. Moreover, H19 can collaborate with BcL2 and nuclear receptor ShP to promote the progression of cholestatic liver fibrosis in rodents (Zhang et al. 2016). Therefore, H19 may be a potential therapeutic target for intervention of some inflammatory and fibrotic diseases.

## Conclusions

H19 is an important oncogenic factor promoting the malignant behaviors of PDAC and other malignant tumors in humans by sponging specific miRNAs in a tumor origin-dependent manner. Furthermore, H19 also regulates inflammation, oxidative stress, and fibrosis by targeting specific miRNAs in different models of inflammatory or fibrotic diseases. Hence, H19 may be a valuable biomarker for the diagnosis and prognosis of PDAC and a promising therapeutic target for the intervention of these diseases. The safety and efficacy of H19-based therapies remain to be demonstrated in clinical trials.

## Data Availability

Not applicable.

## Abbreviations

PDAC: Pancreatic ductal adenocarcinoma
lncRNAs: Long non-coding RNAs
IPMN: Intra-ductal papillary-mucinous neoplasm
EMT: Epithelial-mesenchymal transformation
DTA: Promoter-controlled diphtheria toxin A
EUS: Endoscopic ultrasound
ORs: Odds ratios
CRC: Colorectal cancer
TKIs: Tyrosine kinase inhibitors
HMGA1: High mobility group A
CSCs: Cancer stem cells
LPS: Lipopolysaccharide
PAH: Pulmonary arterial hypertension
EpCAM): Epithelial cell adhesion molecule
ZEB1: E-box binding homologous box 1
VDAC1: Voltage-dependent anion-selective channel 1
COL1A1: Type I collagen alpha 1
